# Does the Coexistence of Food Insecurity and Obesity in Older Adults Explain Variations in Glycated Hemoglobin?

**DOI:** 10.1093/geroni/igaa057.712

**Published:** 2020-12-16

**Authors:** Queendaleen Chukwurah

**Affiliations:** Tulane University, New Orleans, Louisiana, United States

## Abstract

Obesity and food insecurity are known public health concerns for older adults, and both are independent predictors of glycated hemoglobin (HbA1c). I examined the impact of the co-existence of food insecurity and obesity on HbA1c using the National Health and Nutrition Examination Survey (NHANES), 2005-2014. Body mass index /waist circumference (WC) cut- off values were used to create six body types: normal weight with normal WC, overweight with normal WC, obese with normal WC, normal weight with high WC, overweight with high WC, and obese with high WC. HbA1c was defined as normal < 5.7% and abnormal >5.7%. Food security status (FSS) was defined following USDA protocols (food secure-FS, food insecure-FI). The sample population included 5,772 participants 50 years and older with mean (SD) age of 61.8 (0.2). A weighted multivariable logistic regression controlling for age, gender, race/ethnicity, education, and poverty-to-income ratio was run for this analysis. The proportion of older adults with both FI and obesity with high WC (51.1%, p<0.0001) was significantly higher than those FS (37.5%). Logistic regression model with body types and FSS had a maximum-rescaled R-square (MRRS) of 0.147 vs. 0.093 and 0.144 for FSS and body types alone. An increase in MRRS in the model with both body types and FSS compared to the models containing only body types or FSS demonstrates an improved model for fitting abnormal HbA1c levels. The knowledge of this effect may benefit health risk assessment and management in this population.

